# Characterization of the complete chloroplast genome of *Avena chinensis* (Poales: Poaceae)

**DOI:** 10.1080/23802359.2021.1985404

**Published:** 2021-10-05

**Authors:** Hangdong Wang, Jinqing Xu, Xiaolan Li, En You, Lei Wang, Yuhu Shen

**Affiliations:** a Key Laboratory of Adaptation and Evolution of Plateau Biota, Qinghai Provincial Key Laboratory of Crop Molecular Breeding, Laboratory for Research and Utilization of Qinghai-Tibetan Plateau Germplasm Resources, Northwest Institute of Plateau Biology, Chinese Academy of Sciences, Xining, China; b Innovation Academy for Seed Design, Chinese Academy of Sciences, Xining, China; c Scientific Research Station for Modern Agriculture in Wuwei Oasis, Wuwei, China; d University of Chinese Academy of Sciences, Beijing, China

**Keywords:** *Avena chinensis*, chloroplast genome, *Avena*, phylogenetic tree

## Abstract

*Avena chinensis* is recognized as one of the cereals with high nutritional value in the world. In this study, the complete chloroplast (cp) genome sequence of *A. chinensis* was reported. The complete cp genome of *A. chinensis* was 135,899 bp in length with a GC content of 38.51%, including a large single copy (LSC) region of 80,117 bp, a small single copy (SSC) region of 12,576 bp, and a pair of inverted repeated regions of 21,603 bp. The *A. chinensis* cp genome encoded 128 functional genes, including 82 protein-coding genes, 38 tRNAs, and eight rRNAs. The phylogenetic analysis showed that *A. chinensis* was closely related to *Avena hybrid* and *Avena occidentalis*.

*Avena chinensis* (Fisch. ex Roem. & Schult.) Metzg. 1824, an annual herb of the genus *Avena* (Poaceae), is one of the most widely grown cereals in the world and a valuable resource in some countries, both for human consumption and animal feed (Fu et al. 2019). It is mainly distributed in the north, northwest and southwest of China in high latitude, high altitude, alpine arid and semi-arid areas due to its cold-loving, poor and drought-resistant characteristics (Liu et al. 2021). *A. chinensis* belonging to a tribe (Aveneae), separates from the other small-grained cereals such as wheat, barley, rye, triticale (Triticeae) and rice (Oryzeae), which contains 42 chromosomes, representing three distinct sets of nuclear genomes (A, C, and D) (Marshall et al. 2013; Yan et al. 2016). The protein and fat contents of *A. chinensis* are higher than husked oats, nevertheless fiber content is lower (Givens et al. 2004; Biel et al. 2009). In the present study, the complete chloroplast (cp) genome of *A. chinensis* (GenBank accession number: MW784232) was assembled to provide genomic and genetic sources for further research.

The fresh leaves of *A. chinensis* were collected from Huangzhong (101°31′ E, 36°28′ N), Qinghai Province, China. Total genomic DNA of *A. chinensis* was extracted from fresh leaves using the modified CTAB method and quantified (Allen et al. 2006). The voucher specimen and extracted DNA were deposited in the Herbarium of the Northwest Institute of Plateau Biology, Chinese Academy of Sciences (Handong Wang, hdwang@nwipb.cas.cn) under the voucher number WHD2020001. Genome sequencing was performed using the Illumina HiSeq Platform (Illumina, San Diego, CA) at Genepioneer Biotechnologies Inc. (Nanjing, China). Approximately, 26.12 million 150 bp paired-end reads were obtained, and 7.69 GB of clean data was generated after filtering. Then, the clean reads were assembled using SPAdes Version 3.10.1 (Bankevich et al. 2012), and the reference cp genome of *Avena occidentalis* (GenBank accession number: NC_044175.1) was used for quality control after assembly. Finally, the assembled genome was annotated in CpGAVAS (Liu et al. 2012).

The complete cp genome of *A. chinensis* was 135,899 bp in length with a GC content of 38.51%, including a large single copy (LSC) region of 80,117 bp, a small single copy (SSC) region of 12,576 bp, and a pair of inverted repeated regions of 21,603 bp. The *A. chinensis* cp genome encoded 128 functional genes, including 82 protein-coding genes, 38 tRNAs, and eight rRNAs.

The maximum-likelihood phylogenetic tree (ML tree) was generated based on the complete cp genome of *A. chinensis* and 26 other species of the genus *Avena*, with *Oryza sativa* as outgroup, of which the 27 cp genomes for phylogenetic analysis were downloaded from NCBI database. The 28 complete cp genome sequences were aligned by MAFFT v7.037 (Katoh and Standley 2013). The phylogenetic tree was built using MEGA X (Kumar et al. 2018) with bootstrap set to 1000. The phylogenetic tree showed that *A. chinensis* was closely related to *Avena hybrid* and *Avena occidentalis* (Figure 1). This study was the first report on the complete cp genome of *A. chinensis* which could be useful for the phylogenetic and evolutionary studies of *Avena* and Poaceae.

**Figure 1.**
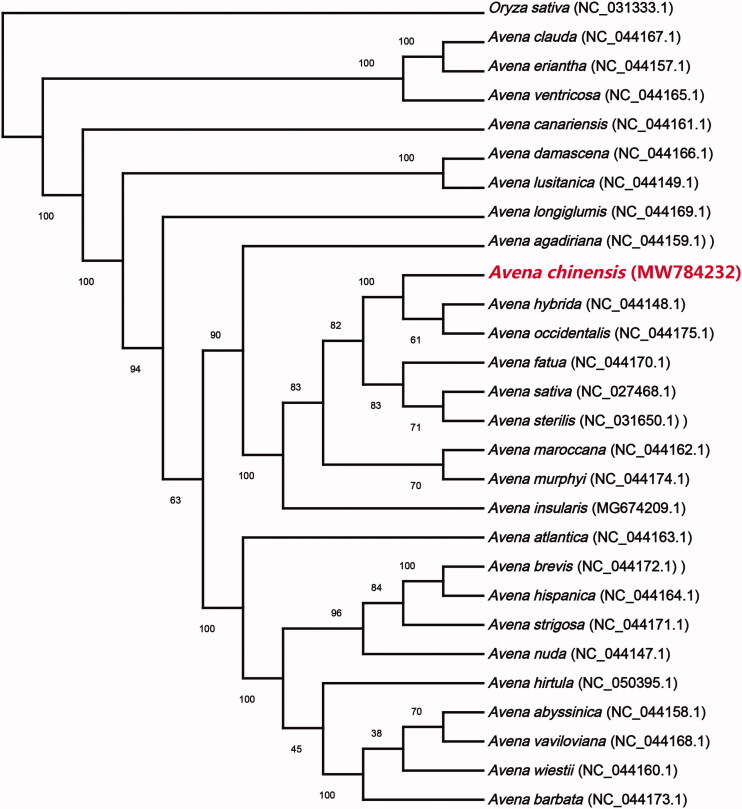
The ML tree based on the complete cp genome of *A. chinensis* and 26 other species of *Avena*, with *Oryza sativa* as outgroup. Numbers below or above the branches indicate the bootstrap value with 1000 replicates.

## Data Availability

The genome sequence data that support the findings of this study are openly available in GenBank of NCBI at https://www.ncbi.nlm.nih.gov/ under the accession no. MW784232. The associated BioProject, SRA, and Bio-Sample numbers are PRJNA743926, SRR15044535, and SAMN20063186, respectively.
